# Some Cases of Cataract Extraction in the Manhattan Eye and Ear Hospital, New York

**Published:** 1871-10

**Authors:** 


### SOME CASES OF CATARACT EXTRACTION IN THE MANHATTAN EYE AND EAR HOSPITAL, N. Y.

REPORTED BY DR. C. A. SIMPSON, OF ATLANTA, GA.

Case I.—William Kelly, æt. fifty-six, was brought to the Hospital in February last, with cataract in both eyes and pterygium of right eye. February 23d, Dr. Roosa performed extraction by an upward flap operation with an iridectomy. The operation was successful, with the result of his being able to read, before leaving, No. 12 of Snellin's types, at nine seconds, and when discharged his vision was equal to 1-10.

September 13th, he applied again at the Hospital to have an operation performed on the other (left) eye. Ether was given, and Dr. Roosa operated by a downward flap. As the flap was being made, the iris fell over the edge of the knife, and was cut. Dr. Roosa then made an iridectomy, to prevent a setting up of iritis, which might have resulted from the bruising of the iris. No vitreous was lost during the operation. Some blood was left in the anterior chamber. The operation terminated very satisfactorily. As soon as the lens was extracted, the eyelids were gently closed, and a retentive band of Liebrich's applied to each eye.

September 14th.—Has had no pain. Bandage carefully removed, and a drop of atropine (solution gr. iv aq. ℥ i) instilled into the corner of the eye without raising lid. The eye was carefully cleansed with tepid water, (there was very little secretion), and bandage renewed.

September 16th.—Removed bandage, cleansed eye and renewed bandage, and instilled atropine without opening eye. No pain; moderate secretion.

September 18th.—Bandage removed, and eye carefully opened in a darkened room. Eye looking well, very little injection, pupil clear; can see objects between himself and window.

September 20th.—Some pain in eye last night. Has a severe attack of asthma. Eye looking well.

September 22d.—Bandage removed and eye examined. Pupil black; very little injection. Counts fingers and recognizes watch. Atropine instilled, and a dark-green shade substituted for the retentive bandage.

October 3d.—Has had a severe attack of bronchitis, from last note up to this time. He also has organic disease of the heart, with systolic murmur, and very irregular heart action. Removed to the Northeastern Dispensary.

Left eye vision equals 2-7 with plus 4 for distance, and with plus 2½ reads Jaeger No. 11 at one foot.

Case II.—Mary C——, æt. sixty-three; cataracta senilis both eyes.

October 6th.—Dr. Agnew operated by the peripheric linear section upward, with Von Græfe's narrow knife, as modified by Dr. Noyes, of this city. Anæsthetic used—ether iv, chloroform i. No vomiting. Section nearly linear, commencing half a line in sclerotica, and from central one-third encroaching on clear cornea. An iridectomy was made, and lens extracted without difficulty. Some bleeding into anterior chamber. Very little corneal matter left. Atropine (solution gr. iv aq. ℥ i) instilled, and Dr. Agnew's black silk dressing applied.

October 7th.—Last night complained of pain in back, both eyes, head and jaws. Gave an opiate, which procured a good night's rest. This morning, complains again of her back. Allowed to sit up in bed; nauseated, and ordered whisky and milk.

October 8th.—Very restless; complains of pain all over, but specially in eyes. Raised corner of dressing and instilled atropine (iv gr. solution); at ten A.M., hypodermic injection of morphine, and leeches to the temple, which relieved the pain.

October 10th.—Removed the dressing yesterday P.M., and instilled atropine after cleansing the eye and renewing the dressing. Has had considerable pain last night and this morning, which was relieved by hypodermic injection of morphine. Atropine instilled.

October 11th.—Eye opened this morning for the first time. Wound healing. Cornea clear; moderate amount of circumcorneal injection. ℞—Atropine ter die, and shade substituted for dressing.

October 12th.—Had considerable pain last night, which was relieved by a hypodermic injection of min. xij of Magendie's solution.

October 14th.—Some iritis. One leech ordered.

October 16th—Much better; counts fingers.

October 21st.—Ordered colored glasses, and allowed to walk about the house.

CASE III.—William H——, æt. fifty-eight; senile cataract.

October 9th.—Dr. Roosa operated by a flap with an iridectomy on right eye. Ether given; no vomiting; no accident during operation. Atropine dropped into eye, and the retentive bandage applied.

October 10th.—Slight pain last night, and flashes of light half a dozen times; thinks it was caused by moving the eye. Eyes carefully cleansed, without opening them; atropine instilled, and bandage renewed. Very little secretion or puffiness of the lids; patient very quiet; slept at intervals last night; appetite good.

October 11th.—Constant pain last night. Dressing renewed. Considerable muco-purulent secretion. Atropine instilled, and again at night.

October 12th.—Pain less; not so much secretion. Bandage to be renewed, and atropine instilled thrice daily. Slight swelling of lids.

October 14th.—Considerable pain last night. Eye opened, and found that there was ulceration of cornea. Atropine ordered to be instilled several times a day, and mix. of tinct. ferri muriat ter die.

October 16.—Eye looking better. Has some pain at night.

October 22.—Improving, and ordered out-door exercise; can recognize objects. A small portion of the cornea is left clear, and his sight may be much improved by an iridectomy.

Case IV.—Thomas H——, æt. seventy-five; senile cataract both eyes; with right eye counts fingers at a distance of one foot; with left eye (pupil dilated by atropine) counts fingers at six inches.

October 13.—Dr. Agnew operated on right eye by a peripheric linear section upwards, with Noye's modified knife. Lens of a dark chocolate color. No vitreous lost, and no accident during operation. Patient was very feeble, and for twenty-four hours after operation, was ordered whisky ℥i every three hours.

October 14th.—Has had constantly decreasing pain since the operation. Slept without an anodyne. Has had considerable hemorrhage from eye, running down and forming clots on neck of patient. Bandage removed, and eye cleansed without opening the lids. New bandage and atropine. Dr. Agnew thought there was detachment of the retina, and that the bleeding was from the choroid.

October 15th.—Pain less; some puffiness of lids. Dr. Agnew's dressing removed, and retentive (Liebrich's) bandage applied. Appetite continues good.

October 18th.—Bleeding continues from eye, but no pain. Bandage reäpplied only to the eye upon which the operation was performed.

October 24th.—Bleeding still continues from eye, and eye is becoming atrophied. No pain.

Case V.—J. F——, æt. forty-three; cataract right eye. Dr. Agnew had operated, some months before, on left eye for cataract.

October 18th.—Dr. Agnew now operated on right eye, by the peripheric linear section downwards. No anæsthetic given, as he could not be gotten under the influence of an anæsthetic for the former operation, and his struggles complicated the operation, and caused the loss of a considerable amount of vitreous. After section with Noye's modified knife, the speculum was withdrawn. Having lacerated the capsule of the lens, by the most careful manipulation, it could not be expelled without great danger of the loss of a considerable quantity of vitreous, from rupture of the hyaloid membrane; whereupon the spoon was introduced beneath the lens, and it extracted, with the loss of only a few drops of vitreous. Atropine, and the retentive bandage applied.

October 19th.—Complained of involuntary jerking and twisting of eye last night. Codia gr. ss. given, and he slept well.

October 20th.—No pain. Appetite good.

October 21st.—Slight pain last night. Renewed bandage; cleansed and opened eye. Cornea clear; no œdema of lids. Atropine instilled, and bandage reäpplied.

October 23d.—Cornea clear; wound healed. Pupil dilated, but cortical matter in field of pupil, giving it a milk-white appearance. The cortical substance is undergoing absorption, and he will likely have useful vision.

Case VI.—J. McD——, aet. thirty-five; cataract both eyes, with only perception of light.

October 18th.—Dr. Agnew operated by peripheric linear section upwards. Anæsthetic—ether j. to chloroform iv. The lens was of a tough, pasty character, and was extracted with some difficulty, although the section was amply sufficient. The spoon was used to complete the delivery. Atropine instilled, and the retentive bandage applied. A considerable amount of cortical matter was left in the pupil.

October 19th.—Considerable pain last night; none to-day.

October 20th.—Complains of much pain in back and bowels. Ordered codia gr. ss.; vomited. Then gave bromid potass. gr. xx., which was soon vomited. Slept some; no pain in eye.

October 21st.—Allowed to eat some rare steak this morning. No pain. Bandage renewed, and eye cleansed and opened. Cornea clear; no swelling of lids. Atropine, and bandage reäpplied.

October 23d.—Cornea clear; pupil black. Black silk dressing substituted for the retentive bandage, and atropine three times daily.

October 24th.—Doing well. Can see objects plainly in the room.

